# Rituximab-induced functional cure of chronic hepatitis B in a patient with autoimmune hemolytic anemia: a rare case report

**DOI:** 10.1093/gastro/goag085

**Published:** 2026-10-14

**Authors:** Lili Wu, Fengmin Lu, Xinhua Li

**Affiliations:** Department of Infectious Diseases, Guangdong Key Laboratory of Liver Disease Research, The Third Affiliated Hospital of Sun Yat-Sen University, Guangzhou, Guangdong, 510630, P. R. China; Department of Microbiology and Infectious Disease Center, School of Basic Medical Sciences, Peking University, Beijing, 100871, P. R. China; Department of Infectious Diseases, Guangdong Key Laboratory of Liver Disease Research, The Third Affiliated Hospital of Sun Yat-Sen University, Guangzhou, Guangdong, 510630, P. R. China

## Introduction

Achieving a functional cure (hepatitis B surface antigen [HBsAg] loss) for chronic hepatitis B (CHB) remains elusive when using standard nucleos(t)ide analogues (NAs) therapy alone [1]. Conversely, B-cell depletion therapy with rituximab (anti-CD20) for autoimmune diseases carries a high risk of life-threatening hepatitis B virus (HBV) reactivation [2]. Here, we report a paradoxical case in which rituximab, administered under intensified antiviral prophylaxis, did not trigger reactivation, but was followed by sustained functional cure in a patient with CHB and refractory autoimmune hemolytic anemia (AIHA). This suggests that controlled B-cell depletion, when combined with potent viral suppression, may unlock an immunological window for viral clearance.

## Case report

A 35-year-old woman with hepatitis B e antigen (HBeAg)-negative CHB presented in 2018 with severe AIHA. The diagnosis of AIHA was confirmed by the presence of reticulocytosis, hyperbilirubinemia, positive Coombs’ and Ham’s tests, and the bone-marrow-biopsy exclusion of other malignancies. Following the failure of cyclosporine and corticosteroids to control hemolysis, we initiated salvage therapy with rituximab. The patient received a standard induction course of rituximab (600 mg weekly for four doses) from 27 June to 17 July 2019. Prednisone was gradually tapered and was completely discontinued on 20 July 2019 (Fig. 1); thereafter, hemolysis remained well controlled until May 2021.

At baseline, the patient had HBeAg-negative CHB and was receiving long-term entecavir (ETV) therapy. However, longitudinal monitoring revealed incomplete viral suppression under ETV monotherapy. In April 2019, the patient developed alanine aminotransferase (ALT) elevation accompanied by low-level viremia (HBV DNA 197 IU/mL) (Fig. 1). Given the concurrent active hemolysis, this liver injury was considered more likely to be related to hemolysis-associated hepatic stress rather than overt HBV reactivation. Nevertheless, to suppress residual viral activity and minimize the risk of subsequent reactivation during intensified immunosuppression, the antiviral regimen was strengthened. Tenofovir disoproxil fumarate (TDF) was added to the ETV in June 2019 to ensure maximal suppression of viral polymerase activity. Under the dual-NA “protective umbrella,” the typical surge in viral replication was effectively blocked. Although a transient, low-level HBV DNA fluctuation was observed at around the time of the fourth rituximab infusion, this did not evolve into sustained viral rebound and may have reflected immunotherapy-associated viral kinetic changes or a hepatocyte injury-related release of HBV DNA-containing material rather than renewed productive replication. The HBV DNA subsequently returned to undetectable levels and, after stabilization of the virological parameters, the TDF was discontinued in August 2019. The patient then resumed ETV monotherapy, with no further HBV DNA fluctuation observed after the TDF withdrawal.

Two months after completion of the first four-dose course of rituximab, HBeAg transiently reappeared during the ALT recovery phase. ALT elevation had already been present before HBeAg became detectable and HBV DNA remained undetectable throughout this period.

Subsequently, the HBsAg levels declined precipitously (<20 IU/mL by August 2020) and ultimately reached seroclearance (<0.05 IU/mL) (Fig. 1). Although the patient experienced a relapse of AIHA in May 2021 requiring a second course of rituximab, the HBV control was maintained. Based on the sustained loss of HBsAg and persistent undetectability of HBV DNA, the ETV was discontinued in January 2022 under extensive surveillance. The patient has since been followed for ∼4 years. The last comprehensive examination at our center was conducted in July 2024, confirming sustained functional cure. Subsequent monitoring was performed via an internet hospital platform in March 2025. Most recently, a telephone follow-up in December 2025 confirmed that the patient remained in good general condition with no signs of hepatitis activity or AIHA recurrence. Serial monitoring throughout this period has consistently demonstrated undetectable HBV DNA and HBsAg levels of <0.05 IU/mL, confirming functional cure. This outcome highlights the durability of the immunological reset achieved.

## Discussion

The mechanism underlying this paradoxical cure is likely multifactorial. Rituximab did not act as a direct antiviral agent, but may have indirectly reshaped the host immune landscape under potent NA coverage. First, a depletion of regulatory B cells, which suppress antiviral T-cell responses in CHB, may have released inhibitory constraints on HBV-specific T cells and facilitated the immune-mediated clearance of infected hepatocytes [3]. Second, chronic HBV infection is associated with dysfunctional HBV-specific atypical memory B cells, characterized by impaired B-cell receptor signaling and defective differentiation into antibody-secreting plasma cells [4, 5]. Transient depletion of this antigen-imprinted compartment may have allowed the reconstitution of a naive B-cell pool with greater potential for humoral immune recovery under sustained viral suppression [6, 7]. This concept is reminiscent of immune replacement after bone marrow transplantation from an HBV-immune donor [8].

Potent NA coverage was probably critical. Antiviral prophylaxis, that initially intensified with ETV/TDF and was subsequently maintained with ETV, may have prevented de-novo viral spread during B-cell depletion and immune reconstitution. In this context, rituximab could function as an immunological reset; without antiviral protection, the same intervention would more likely promote HBV reactivation. The transient HBeAg seroreversion, occurring without HBV DNA breakthrough and during the declining phase of ALT elevation, further argues against typical replication-driven reactivation. This “sterile seroreversion” phenomenon may instead reflect immune-mediated injury or lysis of HBV-infected hepatocytes, leading to the release of sequestered viral antigens. One possible explanation is cross-reactivity between HBeAg and core protein-containing nucleocapsids released from lysed hepatocytes [9]. In addition, retained PreC mRNA transcriptional activity, which may persist despite the NA-mediated suppression of HBV DNA, could permit HBeAg release upon hepatocyte lysis, consistently with the observation that HBV DNA suppression can precede the clearance of hepatitis B core-related antigen and HBsAg [10].

This interpretation remains hypothesis-generating. Because this case was identified retrospectively, peripheral blood mononuclear cells and serum were not prospectively cryopreserved, precluding T-cell phenotyping, cytokine profiling, and CK18-M30/M65 testing [11, 12]. Nevertheless, the sequence of ALT elevation without HBV DNA breakthrough, transient HBeAg reappearance, and subsequent HBsAg clearance supports, but does not prove, an immune-mediated clearance event rather than virological failure.

In conclusion, this case provides a hypothesis-generating rationale that transient anti-CD20-mediated B-cell depletion, under profound viral suppression and close virological monitoring, may create an immunological reconstitution window that restores antiviral immune control in selected circumstances. This interpretation should not be viewed as a clinical recommendation, but rather as a basis for future prospective studies investigating controlled B-cell modulation as a potential adjunctive strategy toward HBV functional cure.

**Figure 1:**
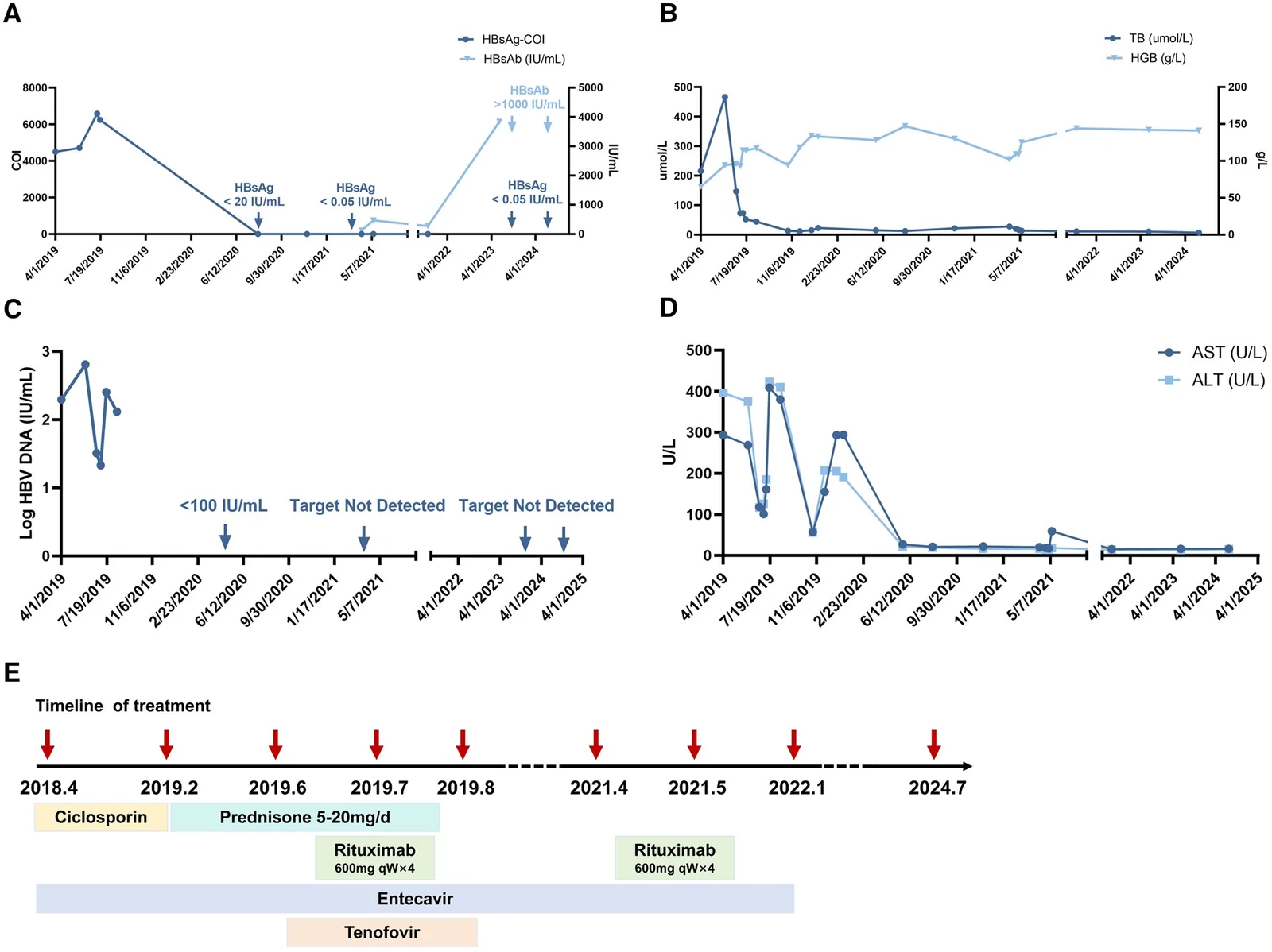
Longitudinal monitoring of clinical parameters and therapeutic timeline. (A) Fluctuations in quantitative HBsAg and (hepatitis B surface antibody) HBsAb levels during the follow-up period. (B) Changes in total bilirubin (TB) and hemoglobin (HGB) indicating the progression of AIHA. (C) Dynamics of Log HBV DNA (IU/mL) viral load over time. (D) Variations in liver enzymes, specifically aspartate aminotransferase (AST) and alanine aminotransferase (ALT). (E) Timeline of therapeutic interventions, showing the duration of rituximab, corticosteroids, and antiviral treatments. Arrows indicate the time points corresponding to the plotted measurements and do not denote additional therapeutic interventions.

## References

[1] Ghany MG , Pan CQ, Lok AS et al AASLD ISDA Practice Guideline on treatment of chronic hepatitis B. Hepatology 2026;83:974–97. 10.1097/HEP.0000000000001549 41186418

[2] Kuo MH , Tseng CW, Ko PH et al HBV reactivation in HBsAg-/HBcAb + rheumatoid arthritis patients receiving biologic/targeted synthetic DMARDs. Liver Int 2024;44:497–507. 10.1111/liv.15793 38010984

[3] Liu Y , Luo Y, Zhu T et al Regulatory B cells dysregulated T cell function in an IL-35-dependent way in patients with chronic hepatitis B. Front Immunol 2021;12:653198. 10.3389/fimmu.2021.653198 PMC8072152 33912178

[4] Burton AR , Pallett LJ, McCoy LE et al Circulating and intrahepatic antiviral B cells are defective in hepatitis B. J Clin Invest 2018;128:4588–603. 10.1172/JCI121960 PMC6159997 30091725

[5] Zhang JW , Lai RM, Wang LF et al Varied immune responses of HBV-specific B cells in patients undergoing pegylated interferon-alpha treatment for chronic hepatitis B. J Hepatol 2024;81:960–70. 10.1016/j.jhep.2024.06.033 38992769

[6] Shao W , Wang Y, Fang Q et al Epigenetic recording of stimulation history reveals BLIMP1-BACH2 balance in determining memory B cell fate upon recall challenge. Nat Immunol 2024;25:1432–44. 10.1038/s41590-024-01900-2 38969872

[7] Gu S , Tang L, Guo L et al Circulating HBsAg-specific B cells are partially rescued in chronically HBV-infected patients with functional cure. Emerg Microbes Infect 2024;13:2409350. 10.1080/22221751.2024.2409350 PMC11523254 39470771

[8] Ilan Y , Nagler A, Adler R et al Ablation of persistent hepatitis B by bone marrow transplantation from a hepatitis B-immune donor. Gastroenterology 1993;104:1818–21. 10.1016/0016-5085(93)90664-x 8500741

[9] Wang J , Huang H, Liu Y et al HBV genome and life cycle. Adv Exp Med Biol 2020;1179:17–37. 10.1007/978-981-13-9151-4_2 31741332

[10] Tseng TC , Chiang C, Liu CJ et al Low hepatitis B core-related antigen levels correlate higher spontaneous seroclearance of hepatitis b surface antigen in chronic hepatitis B patients with high hepatitis B surface antigen levels. Gastroenterology 2023;164:669–79.e666. 10.1053/j.gastro.2023.01.005 36642151

[11] Cao ZJ , Li J, Wang Y et al Serum hepatocyte apoptosis biomarker predicts the presence of significant histological lesion in chronic hepatitis B virus infection. Dig Liver Dis 2016;48:1463–70. 10.1016/j.dld.2016.07.037 27575659

[12] Papatheodoridis GV , Hadziyannis E, Tsochatzis E et al Serum apoptotic caspase activity as a marker of severity in HBeAg-negative chronic hepatitis B virus infection. Gut 2008;57:500–6. 10.1136/gut.2007.123943 18025069

